# Screening for paediatric sleep disordered breathing in the dental setting: a scoping review

**DOI:** 10.1007/s11325-026-03646-7

**Published:** 2026-03-20

**Authors:** Raksha Baskar, Moya Vandeleur, Emily Trinh, Mihiri Silva

**Affiliations:** 1 Melbourne Dental School, University of Melbourne, Carlton, VIC Australia; 2 Murdoch Children’s Research Institute, Parkville, VIC Australia; 3 Dept. of Respiratory and Sleep Medicine, the Royal Children’s Hospital, Melbourne, Australia

**Keywords:** Pediatric dentistry, Sleep apnea syndromes, Snoring, Scoping review

## Abstract

**Background:**

Sleep-disordered breathing (SDB) is under-recognised and under-diagnosed in children. Therefore, SDB screening during routine dental appointments may be considered a scalable approach to identifying at-risk children and guiding referrals to sleep specialists. This scoping review aims to explore the literature on paediatric SDB screening in dental settings and identify knowledge gaps.

**Methods:**

A scoping review was conducted using Medline, Embase, and PubMed. The inclusion criteria were: studies conducted in dental settings that describe SDB screening in non-syndromic patients aged 18 or younger, without previous surgical management for OSA.

**Results:**

Thirty-four studies were included, most of which were observational descriptive studies (*n* = 32). Eleven screening tools were used across studies: the most common being the Paediatric Sleep Questionnaire (*n* = 23). Numerous studies (*n* = 16) have reported the prevalence of positive SDB screening outcomes among the paediatric population, while others explored associations with sociodemographic factors (*n* = 7), craniofacial features (*n* = 11), medical conditions (*n* = 9), orthodontic treatment (*n* = 6), and oral health outcomes (*n* = 4). Two studies developed novel screening measures. A small number of studies discussed referral pathways (*n* = 7), and only one study documented referral outcomes.

**Conclusions:**

This review highlights the need for further validation of paediatric sleep questionnaires within dental settings. However, additional validation of clinical assessment tools in this context may offer limited incremental benefit. Furthermore, none of the included studies examined how screening protocols integrate within routine dental workflows or explored perceptions of children, caregivers, and dental practitioners. Lastly, there is a paucity of evidence concerning referral pathways and follow-up outcomes subsequent to screening.

**Supplementary Information:**

The online version contains supplementary material available at 10.1007/s11325-026-03646-7.

## Introduction

Sleep-disordered breathing (SDB) encompasses a spectrum of conditions, ranging from primary snoring (PS), upper airway resistance syndrome, obstructive hypoventilation to obstructive sleep apnoea (OSA) [1]. PS involves habitual snoring, without apnoeas, hypopneas, gas exchange abnormalities, or arousals on polysomnography (PSG) [2]. OSA is characterised by prolonged partial or intermittent complete upper airway obstruction, accompanied by symptoms such as snoring, witnessed apnoeas, restless sleep, morning headaches, and daytime dysfunction [2–4]. Sleep-disordered breathing is associated with adverse neurobehavioural, metabolic, and cardiovascular outcomes [5, 6]. The prevalence in children is 2–11%, peaking between ages 2 and 7, with 8–27% experiencing frequent snoring and 1–5% affected by OSA [1, 7, 8]. The gold standard for definitive diagnosis of SDB is an overnight PSG [9]. 

Sleep-disordered breathing remains underdiagnosed in the paediatric population, with diagnosis frequently delayed [10, 11]. This is thought to be due to the high cost and limited accessibility to testing services, as well as poor caregiver awareness of sleep problems which leads to underreporting of symptoms to medical practitioners [12–14]. Early identification and management of SDB is crucial in light of the significant negative impacts on health [15]. 

Screening questionnaires such as the Paediatric Sleep Questionnaire (PSQ) have been designed to identify patients with suspected SDB. They represent a simple and cost-effective way to facilitate early identification of SDB, by screening patients at risk and triaging those who would benefit from referral to sleep specialists [16, 17]. Unlike polysomnography, screening questionnaires are limited to evaluating SDB risk and lack the diagnostic accuracy needed for a definitive diagnosis [18]. 

Due to the significant prevalence of SDB in paediatric populations and its association with adverse health outcomes, including reported links to oral health, dental appointments may represent an opportunity for community screening of SDB [19]. This potential value is supported by the high level of routine attendance among children, with approximately 80% of children in Australia reported to visit their dentist annually [20]. Adult cross-sectional studies indicate good levels of patient acceptance of screening for medical conditions in dental settings. Factors including younger age, positive relationship with the dentist, perceived dentist expertise, and prior screening history are associated with favourable attitudes toward screening [21–24]. Professional associations like the American Dental Association (ADA) and the American Academy of Paediatric Dentistry (AAPD) encourage dentists to screen all patients for SDB during routine dental visits and recommend referral to specialists such as otolaryngologists, sleep medicine physicians, or pulmonologists for further assessment when SDB is suspected [5, 25, 26]. A pilot survey of paediatric dentists in the USA found that SDB screening was not standard practice, with only 40.7% routinely screening for SDB [27]. 

While paediatric sleep-disordered breathing screening in dental settings has been investigated, the existing literature is incomplete and lacks comprehensive integration. A recent narrative review described the role of dentists in SDB screening, but it was not child-specific and provided a broad overview of screening and clinical examination techniques utilised in both medical and dental settings, rather than mapping empirical evidence on SDB screening in dental practice [28]. Furthermore, most prior systematic reviews have focused on non-dental settings, such as sleep centres and hospitals, or on screening adult patients in dental practice [16, 17, 29]. Therefore, the aim of this scoping review is to map and describe the extent and characteristics of current evidence around paediatric SDB screening in dental settings and identify key evidence gaps to inform future clinical research. A scoping review was undertaken as it was more suitable for examining the breadth of current evidence, rather than answering a clinical question, which would have required a systematic review.

## Methods

**Fig. 1:**
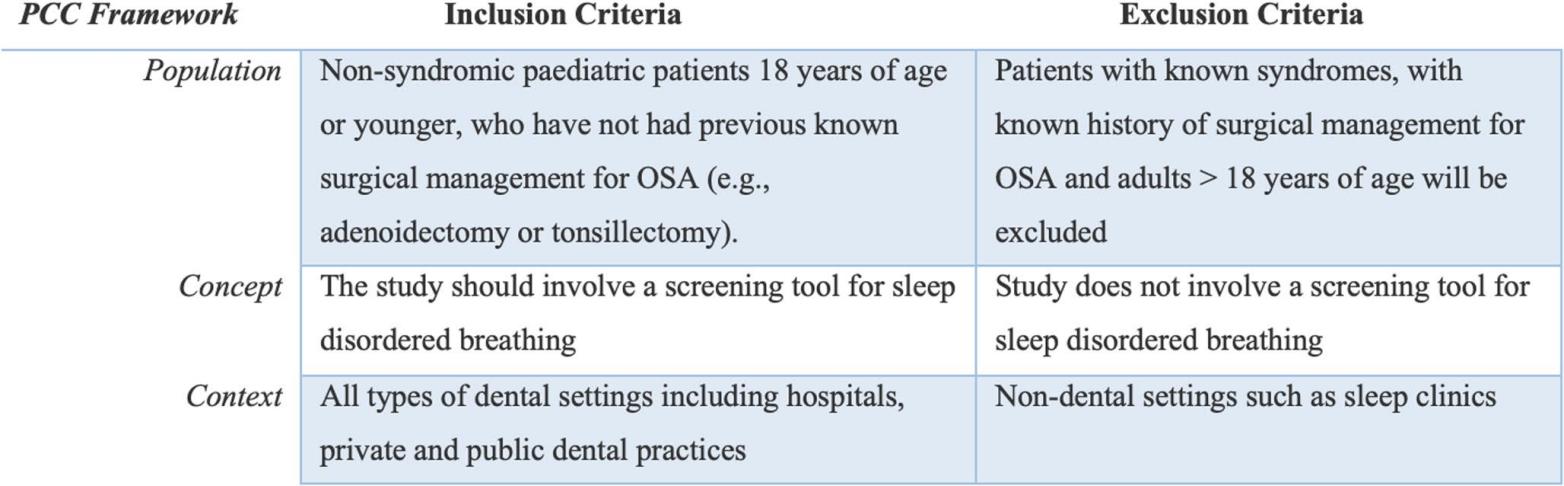
Eligibility Criteria

This scoping review followed Joanna Briggs institute methodology, using the population (paediatric patients), concept (screening tools) and context (dental settings) framework to develop the review question and eligibility criteria (Fig. 1) [30]. The primary review question was: *What is the scientific evidence for screening paediatric patients for sleep-disordered breathing in a dental setting?* The objectives of this review were to identify and describe the studies conducted on screening children for SDB in a dental setting, and to highlight gaps in the literature. The inclusion criteria were studies describing SDB screening tools in non-syndromic paediatric patients 18 years of age or younger who have not had known surgical management for OSA (i.e. adenoidectomy or tonsillectomy). This review included studies conducted in dental settings including hospitals, private and public dental practices. No restrictions were placed on publication date, language, or country. Opinion papers, unpublished studies, comments, and letters were excluded. The scoping review protocol was registered on OSF on the 15th April 2024 (Baskar, 2024).

### Search strategy

This study adhered to PRISMA-ScR guidelines (Supplementary Figure S1). A comprehensive literature search was conducted in April 2024 across Medline, Embase, and PubMed using broad search terms related to questionnaires, sleep-disordered breathing, dentists, and children (Supplementary Figure S2). Following the initial search, all potentially eligible articles were uploaded into Covidence and duplicates were removed. Two reviewers (RB and ET) independently screened the title and abstracts of all potentially eligible studies and assessed their eligibility for inclusion based on a full-text review. Any disagreements between reviewers were resolved through discussion.

### Data extraction

A data extraction tool was developed to record the relevant information related to the research question, including: (1) bibliographic details such as author, year of publication, country of origin; (2) study characteristics including aims/objectives, design, population, sample size, outcome variables and (3) findings relevant to the review question. The screening tools were further described according to target age, number of questions, components, respondent, and the scoring system. Data extraction was performed by the primary reviewer (RB), and a random 10% sample was audited by a second reviewer (ET), with no discrepancies identified.

## Results

**Fig. 2:**
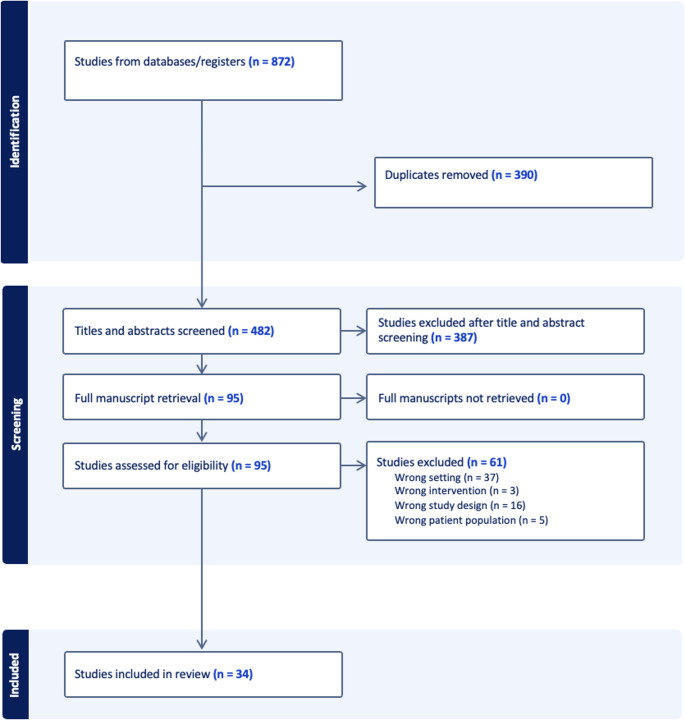
PRISMA Flowchart

The initial search yielded 872 articles, which was reduced to 482 after removing duplicates. Following the screening of titles and abstracts against inclusion criteria, 95 potentially eligible articles were included for full text-review. A further 61 further articles were excluded after full-text review, resulting in a total of 34 articles included in the final analysis. (Figure 2).

### Study characteristics

**Fig. 3:**
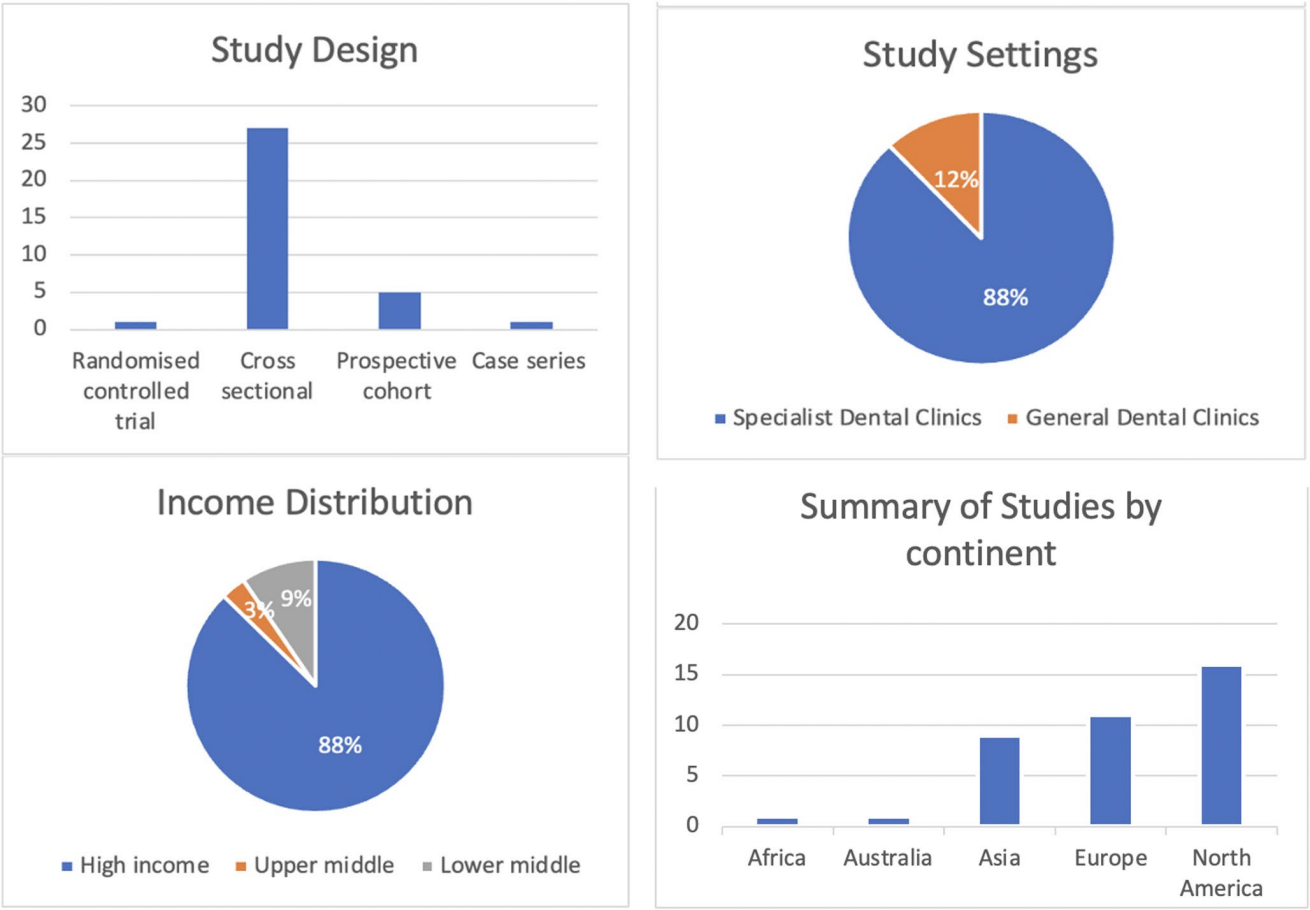
Study Characteristics

Figure 3 summarises the study characteristics. Most included studies had an observational descriptive study design, consisting of mainly cross-sectional (*n* = 27) and prospective cohort studies (*n* = 6) with one randomised controlled trial included in the sample. Only one of the articles utilised a qualitative approach [31]. Majority of the articles were published in high-income countries (*n* = 28), such as the USA (*n* = 10), Italy (*n* = 6), Canada (*n* = 5), Australia (*n* = 1), Hong Kong (*n* = 1), Germany (*n* = 1), Spain (*n* = 2), South Africa (*n* = 1), Turkey (*n* = 2), UAE (*n* = 2), South Korea (*n* = 1), and Japan (*n* = 1). The remaining articles were published in upper-middle-income and lower-middle-income economies, such as India (*n* = 3), Malaysia (*n* = 1), Mexico (*n* = 1), and Thailand (*n* = 1). Most articles were published after 2020 (*n* = 22), with 13 published after 2022. The earliest article was published in 2001, and the most recent in 2024. Many studies included in the review were single-country, single-centre studies with only nine multicentre and three multinational studies included.

### Participants

The age of participants in the studies ranged from 2 to 18 years. However, the two studies did not disclose the exact age range; instead, they reported their samples as including children or paediatric patients [32, 33]. Eleven studies recruited patients under the age of 12 only. There was a wide range in the number of participants in the study, from 16 to 1209. Most studies had a balanced sex distribution, with male participants comprising between 39% and 60% of the total sample. Some studies excluded participants who had prior orthodontic treatment (*n* = 11), however, others recruited patients who specifically had a malocclusion (*n* = 5) or who were currently undergoing orthodontic treatment (*n* = 2). Several studies (*n* = 18) excluded participants with medical conditions or craniofacial abnormalities. Most studies recruited participants from university-teaching dental clinics (*n* = 23), with the remainder of participants recruited from private practices (*n* = 8) or hospital dental departments (*n* = 3). Many studies were conducted in specialist orthodontic (*n* = 17), or paediatric dental settings (*n* = 13), with few studies conducted in general dental clinics (*n* = 4).

## What is the scientific evidence on screening paediatric patients for sleep-disordered breathing (SDB) in a dental setting?

### Screening tools

In total, 11 screening tools were used across the studies. The PSQ was the most used screening tool, featured in 23 studies in various languages, including English, Turkish, Spanish, Arabic, Chinese, and Italian. Positive PSQ responses are scored as a proportion of the total responses, with a score greater than 0.33, or more than 7 positive answers, indicating a risk of SDB [34]. Following the PSQ, the Sleep Disturbance Scale for Children (SDSC) (*n* = 4) and the OSA-18 (*n* = 2) were the next most utilised tools. The OSA-18 was developed to assess the quality-of-life impact of paediatric SDB [35]. Both the SDSC and OSA-18 use graded scales summed into a total numerical score, to determine whether SDB risk is present [33, 36–38]. Other screening tools included the Children’s Sleep Habits Questionnaire (CSHQ) (*n* = 1) and Pittsburgh Sleep Quality Index (*n* = 1), scored with numerical values. However, the CSHQ evaluates general sleep domains rather than serving as an SDB-specific screening tool, while the Pittsburgh Sleep Quality Index is validated only for adults aged 19–80 years [39–41]. Some studies used lesser-known questionnaires, including those developed by the authors for dental settings, such as the sleep questionnaires by Bergersen (2015) (*n* = 1), Yerra and Shetty (2021) (*n* = 1), and Ali et al., (1993) (*n* = 1). One study failed to provide details about the questionnaire used [42]. Clinical assessment scores were also utilised in few studies including tonsil size and Mallampati score (*n* = 1), FAIREST-15 (*n* = 1) and FAIREST-6 (*n* = 1). Table 1 summarises the screening tools and frequency of use across the studies.

**Table 1:** Summary of Screening Measures

Screening Tool	# Studies	Age	# Questions	Components	Scoring System	Respondent
Paediatric Sleep Questionnaire [34]	23	2 to 18	22	3 symptom complexes: snoring, excessive daytime sleepiness, inattentive or hyperactive behaviour	Proportion of positive scores (> 33% indicates risk)	Parent/caregiver
Sleep Disturbance Scale for Children [37]	4	6 to 15	26	6 subdomains: difficulty initiating and maintaining sleep, sleep breathing disorders, disorders of arousal, sleep-wake transition disorders, disorders of somnolescence, sleep hyperhidrosis.	Likert scale; total score 26–130; cut-off score of 39	Parent/Caregiver
OSA-18 [43]	2	6 months to 12 years old	18	Sleep disturbances, physical symptoms, emotional symptoms, daytime functions, and caregiver concerns	Graded scale; total score above 60 indicates high SDB risk	Parent/Caregiver
Tonsil Size and Mallampati Score	1	11 to 14	NA	Tonsil size and Mallampati score	Tonsil size (Friedman grading scale)	Clinician
FAIREST-6 [44]	1	6 to 12	6 components	Functional (Mouth-breathing), Extraoral (Mentalis strain), Intraoral soft tissue (Tonsil hypertrophy, Ankyloglossia, ) Intraoral hard tissue (Dental wear and Narrow palate)	Score of 2 (mild), 4 (moderate), 6 (severe)	Clinician
FAIREST-15 [45]	1	Not reported	15 items	Clinical assessment (dental, otolaryngologic, and functional risk factors) + six parent reported subjective measures (breathing route, posture, concentration, and anxiety.)	Sum of items	Clinician
Sleep Questionnaire by Yerra & Shetty [46]	1	4 to 10	4 sections	Demographics, medical history, sleep practices, specific sleep problems	None described	Parent/Caregiver
Pittsburgh sleep quality index [47]	1	19 to 80	19	7 components: subjective sleep quality, sleep latency, sleep duration, habitual sleep efficiency, sleep disturbances, use of sleeping medication, and daytime dysfunction.	Sum of items for 7 components. Score > 5 indicates a poor sleeper.	Self
Sleep questionnaire by Ali et al. [48]	1	4–5 years	13	Snoring and daytime sleepiness, hyperactivity, and restless sleep.	None described	Parent/Caregiver
SDB Questionnaire for children by Bergersen [49]	1	< 18	27	Abnormal symptoms of breathing, snoring, hyperactivity, restless sleep, daytime sleepiness and dysfunction.	Scale of severity from 1–5	Parent/Caregiver
Children’s sleep habits questionnaire (CSHQ) [41]	1	4 to 10	45	Sleep onset and bedtime behaviour; duration of sleep; anxiety around sleeping; behaviour observed in sleep and night waking; sleep-disordered breathing; daytime sleepiness and parasomnias.	3-point scoring: Usually, Sometimes or Rarely. Includes a total score as well as a subscale score.	Parent/Caregiver

## Proportion of Children with Positive SDB Risk

Many included studies (*n* = 16) investigated the proportion of children with positive SDB risk within their study population. Among these, 15 studies relied solely on screening questionnaires to assess SDB risk [32]. Though most of these (*n* = 12) accurately indicated that their figures identified patients with questionnaire-determined SDB risk, three incorrectly claimed that participants had SDB, which cannot be definitively diagnosed through questionnaires [33, 50, 51]. Only one longitudinal observational study conducted in Italy utilized respiratory polygraphy and reported that of the 134 patients screened with questionnaires, 28.3% tested positive for OSA with respiratory polygraphy [32]. Estimates of the proportion of children with positive SDB risk varied widely between studies, ranging from 1.1% to 69%. Some studies reported the proportion of patients with questionnaire-reported symptoms such as snoring (*n* = 2) and sleep bruxism (*n* = 1), rather than SDB risk [42, 44, 52]. 

## Craniofacial, medical, sociodemographic risk factors and SDB

Several studies (*n* = 20) described the association between risk factors including craniofacial, medical, sociodemographic, and anatomical characteristics - and SDB risk, as summarised in Table 2. Most studies (*n* = 16) relied on clinical orthodontic assessments, with four also analysing radiographic features using lateral cephalograms [33, 53–55]. Eleven studies explored the association between craniofacial characteristics and SDB risk. Craniofacial characteristics included narrow palate, class II and III malocclusions, retrognathic mandible, convex profile, high mandibular plane angle, dolichofacial growth pattern, crowding, overjet, and retroclined upper incisors [10, 26, 33, 45, 50, 53–59]. Among these, ten were cross-sectional descriptive studies, while one was a longitudinal observational study conducted in a Spanish hospital that examined the link between dentofacial features and questionnaire-determined SDB risk before and after orthodontic treatment [33]. 

**Table 2:** Studies reporting associations between risk factors and SDB risk

Authors	Risk Factors Reported			
	Clinical Airway Indices*	Craniofacial characteristics	Medical Comorbidities	Sociodemographic characteristics
Zreaqat et al. (2021) [60]	✓		✓	
Vazquez-Casas et al. (2020) [33]		✓		
Burnheimer et al. (2023) [61]			✓	✓
Oh et al. (2021) [28]	✓	✓		
Shirke & Katre (2023) [50]	✓	✓		✓
Huynh et al. (2011) [62]	✓	✓	✓	
Kim et al. (2023) [55]		✓	✓	✓
Lesavoy et al. (2022) [63]	✓			
Wellham et al. (2023) [64]				✓
Di Carlo et al. (2020) [1]			✓	✓
Okuji et al. (2020) [26]		✓	✓	✓
Baddam et al. (2019) [65]		✓		
Abtahi et al. (2020) [66]			✓	
Abdalla et al. (2022) [10]	✓	✓	✓	
Duman & Vural (2022) [56]		✓		
Tsuda et al. (2011) [36]		✓		
Rohra et al. (2018) [67]				✓
Blumer et al. (2022) [5]			✓	
Fernandes et al. (2021) [68]		✓		
Graf et al. (2016) [42]		✓.		

*Clinical airway indices: Clinical measurements/ assessments to determine the anatomy of the airway e.g., tonsil size, Mallampati score and turbinate hypertrophy

### Oral Health Outcomes

Few studies (*n* = 4) examined the link between SDB risk and oral health outcomes, in the context of proposed associations between SDB-associated craniofacial features, mouth breathing, xerostomia, and oral disease [69]. Two descriptive cross-sectional studies reported on the association between SDB risk and outcomes such as caries experience, periodontal health, oral health-related quality of life and dental defects [10, 69]. One of these studies compared oral health outcomes between a group with positive SDB risk and another group without risk [69]. In addition, two other cross-sectional studies investigated the relationship between questionnaire-reported SDB symptoms (e.g. mouth breathing, bedtime problems, night awakenings, nocturnal symptoms, and morning symptoms) and parental-reported bruxism [44, 70]. 

### Orthodontic treatment and SDB risk

Some prospective cohort studies (*n* = 5) and one randomised-controlled trial evaluated the relationship between orthodontic treatment and SDB risk [31, 33, 49, 71–73]. Three of these cohort studies used descriptive questions to examine the association between orthodontic treatment and SDB risk, comparing screening questionnaire results before and after treatment [31, 33, 72]. Of these, two found no significant change in SDB symptoms, while one reported improvement following rapid maxillary expansion (RME) [31, 33, 72]. Two studies attempted to address causal questions about the effect of orthodontic treatment, including preformed appliances and twin block appliances, on SDB risk. Though these studies reported an improvement in SDB symptoms, they were both longitudinal observational studies without a control group, making it difficult to control for confounding factors or establish causality [49, 71]. One randomised controlled trial in Hong Kong comparing HG-Herbst and HG-TB treatments found no significant differences between treatment groups in PSQ scores, though scores improved independently in both groups [73]. Three studies specified malocclusion types in their inclusion criteria, such as Class II (*n* = 2) and/or transverse maxillary deficiency (*n* = 1), limiting the applicability of the findings to certain malocclusions [31, 71, 74]. Table 3 summarises these studies.

**Table 3:** Studies reporting relationship between Orthodontic Treatment & SDB Risk

Study Information	Inclusion Criteria	Exclusion Criteria	Type of Orthodontic Treatment	Screening tool used
Batra & Shetty. (2022) [71]	Skeletal class II malocclusion,ANB>5*, AO>BO(>1 mm), SNA 80-83*, retrognathic mandible (SNB<78*), horizontal growth pattern, Stage 3 CVM and good compliance.	Increased vertical growth, anterior open bite, posterior crossbites severe maxillary transverse deficiency, enlarged adenoids and tonsils, mouth breathing, previous orthodontic treatment, chronic allergies, nasopharyngeal obstructions, septum deviations, upper airway surgeries, and cramiofacial anomalies.	Twin-block appliance	Sleep questionnaire Yerra, A & Shetty, V(2021)
Bergersen et al. (2022) [75]	Aged 2 to 13 with one or more of the following symptoms: night-time mouth breathing, daytime oral breathing, snoring, suspected OSA, or bedwetting.	Not meeting inclusion	Preformed habit-modifying appliance( Healthy Start Habit Corrector Appliance)	SDB Questionnaire for children by Bergersen et al. (2015)
Changsiripun et al. (2019) [72]	No medical disorders	Poor compliance, parents unable to obeserve sleep habits, questionnaires were completed by different caregivers.	Orthodontic removable appliances with posterior bite planes	OSA-18 and PSQ
Helal et al.(2019) [31]	ASA 1 status, 5-13 years, transverse, maxillary deficiency, clinical bilateral posterior crossbite, and parental perception of breathing difficulties.	Not meeting inclusion	Rapid Maxillary Expansion	PSQ
Vazquez-Casas et al.(2020) [33]	No craniofacial malformations and/or respiratory and neurological disease	Craniofacial malformations and/or respiratory and neurological disease	Not Specified	PSQ(Spanish) and SDSC
Gu, Savoldi, Hagg, et al.(2019) [73]	12-17 years old for boys and 10-15 years old for girls (ages at which the pubertal growth spurt occurs), bilateral class II molar relationship, increased incisal overjet(>5 mm)	Cleft lip and palate, craniofacial syndromes, and severe transvere maxillary deficiency	Headgear Herbst (HG-Herbst) or the headgear Twin Block (HG-TB) appliance	PSQ (Chinese)

### Novel methods of screening

A small number of studies (*n* = 2) employed existing screening questionnaires to develop and validate novel methods for screening SDB in the dental setting. One prospective cohort study in Mexico used the PSQ to validate a novel mobile application and determine its sensitivity and specificity for detecting primary snoring [76]. Another cross-sectional study identified functional, extraoral, and intraoral features linked to increased SDB risk in children, as determined by the SDSC questionnaire. These features- including mouth breathing, mentalis strain, tonsillar hypertrophy, ankyloglossia, dental wear, and narrow palate, were used to develop the FAIREST-6 clinical screening tool [45]. 

### Follow-up and management

**Fig. 4:**
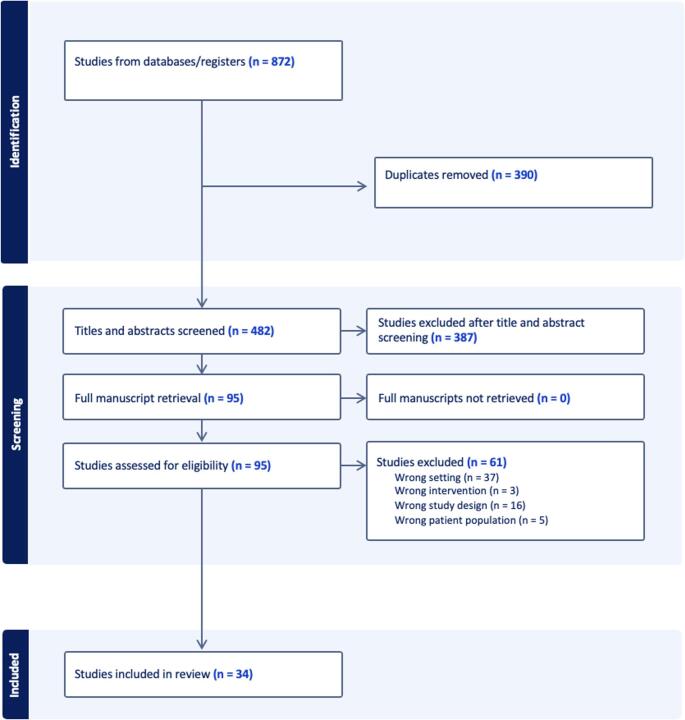
Follow Up Pathways

Only seven of the included studies outlined follow-up actions for patients who screened positive for SDB risk. (Fig. 4) Of these, six described directing at-risk patients to various specialists including paediatric sleep specialists (*n* = 2), sleep clinics (*n* = 1), ENT specialists (*n* = 3), nutritionists (*n* = 1), orthodontists (*n* = 1), and speech therapists (*n* = 1). One study mentioned referral but did not specify the type of practitioner to whom patients were referred. Among these seven studies, six referred all patients identified at-risk based on the screening tool’s threshold cut-off for SDB. However, one prospective cohort study in the USA referred at-risk patients only if they exhibited Friedman Stage 3 or 4 tonsillar enlargement or impaired nasal breathing [49]. While most studies did not provide details about referral process, one observational descriptive study conducted in an Italian paediatric and orthodontic clinic outlined a multidisciplinary referral and management pathway. This pathway involved placing all diagnostic suspects on a waitlist for home respiratory polygraphy as well as referral to a paediatrician. If respiratory polygraphy confirmed the presence of OSA, patients were referred to specialists based on suspected aetiology such as dieticians for obesity, otolaryngologists for nasal obstruction, orthodontists for malocclusions, and speech therapists for orofacial muscular dysfunction [32]. Most of these studies (*n* = 6) did not report on the outcomes of referrals to medical practitioners.

## Discussion

This scoping review examines the evidence relating to screening children for SDB within dental settings. Most studies were observational and descriptive, utilising cross-sectional or cohort designs. They primarily aimed to identify the proportion of patients at risk for SDB in dental settings and to explore associations with sociodemographic characteristics, craniofacial features, medical risk factors, oral health outcomes, and orthodontic treatment.

Across studies, screening approaches broadly fell into two categories: questionnaires and clinical assessment scores. Most studies used screening questionnaires previously validated in paediatric populations, with the PSQ being the most common [34]. The PSQ offers several advantages for dental use, including its specific focus on paediatric SDB, brevity, simple yes/no/don’t know response format, and widespread use in earlier research [33, 50, 61, 65, 77, 78]. In contrast, the Sleep Disturbance Scale for Children (SDSC) evaluates a broader range of sleep disorders, and the OSA-18 functions primarily as a quality-of-life measure. Both utilise more detailed, graded response formats, which may reduce their practicality for routine screening in dental settings [37, 72, 79]. One systematic review and meta-analysis found that the PSQ demonstrated greater sensitivity than the OSA-18 for detecting paediatric OSA at an AHI threshold of ≥ 1 [17]. 

The objective of our review was not to address specific questions regarding the relationship between SDB and risk factors or the effects of orthodontic treatment on SDB. These questions would be better addressed with a systematic review methodology. Instead, we aimed to broadly map the available evidence on paediatric SDB screening in a dental setting and identify gaps in research.

Based on the findings of this review, several key gaps in the literature were identified. One major gap relates to the validation of screening tools in dental settings. While screening questionnaires like the PSQ are extensively validated in medical contexts, none of the included studies assessed their validity in dental practices [34, 35, 80]. The validity of screening questionnaires in dental settings may be influenced by caregiver reporting biases, as caregivers might underreport symptoms because they perceive the questions as irrelevant in a dental setting or respond favourably to reflect satisfaction with treatment [33, 51, 70, 81, 82]. In contrast to screening questionnaires, the evidence supporting clinical assessment tools (e.g., Mallampati score or tonsil size) in screening for SDB in dental settings is weak. Assessments conducted while the patient is awake may differ from those during sleep due to the dynamic nature of airway anatomy, potentially leading to inaccurate risk assessments [83, 84]. Similarly, tools like the FAIREST-15 and FAIREST-6 scales, which evaluate dental, otolaryngologic, and functional characteristics associated with increased SDB risk, have limited evidence regarding their accuracy and reliability [44, 45]. While these tools may be incorporated into routine clinical examinations, their utility as primary screening instruments for SDB remains uncertain. Therefore, while this review highlights the need for further research validating questionnaires in dental settings, additional validation of clinical assessment tools in the dental context may be of limited benefit.

A second important gap is the lack of research exploring the feasibility and acceptability of SDB screening in dental practices. None of the included studies examined how screening fits into routine dental workflows or how it is perceived by children, caregivers, and dentists. Qualitative research could provide insight into the broader considerations of screening from both caregiver and clinician perspectives, helping to understand the practical challenges and potential for implementing SDB screening in everyday dental practice [85]. 

A third critical gap is the limited evidence on referral pathways and follow-up after screening. Further research on how patients with positive SDB risk were referred to medical practitioners, and to document the proportion of patients who received a definitive diagnosis or further treatment would be beneficial. This would help clarify the clinical utility of dental screening and its role in supporting the medical assessment and management of SDB.

The final gap in the current body of evidence is the lack of studies from low- and middle-income countries. Most studies included in this review were conducted in high-income countries, which limits the generalisability of findings to settings with different healthcare systems, access to dental care, and referral pathways. Research from a broader range of countries is needed to understand how paediatric SDB screening can be implemented effectively across diverse dental care contexts.

Most studies included in this review were observational in design, which may limit the ability to draw causal conclusions especially without appropriate study design to account for bias. However, a few studies attempted to infer causal relationships, particularly between orthodontic treatment and SDB, without being suitably designed for causal inference. Conducting additional observational studies, without addressing these methodological limitations, could contribute to avoidable research waste [86]. Future research should prioritise well-designed studies that include both treatment and comparison groups, to explore whether early orthodontic intervention reduces SDB risk in children. Similarly, although several studies described the proportion of children with SDB risk attending dental practices, the value of further research in this area is uncertain, as numerous studies already address community SDB prevalence [87–92]. Repeating prevalence studies without extending their scope to address follow-up contributes to research waste.

To our knowledge, this is the first scoping review to specifically examine the literature on screening for paediatric SDB within dental settings. While one systematic review has evaluated the evidence for dentists screening patients of all ages for SDB, similar reviews focusing exclusively on children are lacking [29]. Of note, most sources included in that systematic review were guidelines or expert consensus statements from recognised medical or dental organisations not underpinned by high-level clinical evidence [29]. Although associations such as the ADA and AAPD have issued recommendations encouraging dentists to screen for SDB in paediatric populations, this scoping review identified limited empirical evidence on the implementation and effectiveness of dentist led screening to support these professional recommendations [5, 25, 26]. The findings of this review highlight that the existing literature is dominated by observational prevalence studies, with minimal validation of screening tools in dental environments and a marked lack of data on feasibility, acceptability, referral pathways, and post-screening outcomes. By comprehensively mapping this evidence, the review clarifies the true scope and limitations of current knowledge and identifies clear priorities for future research to support evidence-based implementation of paediatric SDB screening in dental practice.

This review has some limitations. Populations such as children with known syndromes and those who had already undergone surgical management for OSA were excluded due to their distinct risk profiles and clinical management considerations [93]. Consequently, the findings primarily reflect screening in the general paediatric dental population and may not be directly applicable to high-risk subgroups. Future studies should specifically examine the role of screening in these populations. Additionally, by restricting the scope of our review to dental settings, we may not have captured a broader range of SDB screening tools utilised in medical settings.

## Conclusion

Despite recommendations from peak dental health bodies for dental professionals to screen regularly for SDB, evidence on the practical implementation of paediatric SDB screening in dental settings remains limited. In this review, the PSQ was the most frequently used screening tool for paediatric SDB in dental settings. However, no studies evaluated the feasibility and acceptability of screening in dental settings from caregiver and clinician perspectives. Furthermore, very few studies document the outcomes of positive screening, including referral, follow-up care and medical management. These findings highlight key knowledge gaps that need to be addressed to inform the development of effective screening and referral workflows in dental practice. 

## Supplementary Information

Below is the link to the electronic supplementary material.

Supplementary Material 1 (DOCX 51.3 KB)

Supplementary Material 2 (DOCX 689 KB)

Supplementary Material 3 (DOCX 65.0 KB)

## Data Availability

All data supporting the findings of this study are available within the article and its supplementary material.
