# Supplementary figures and images for: Screening for paediatric sleep disordered breathing in the dental setting: a scoping review
Source: Sleep Breath. 2026 Mar 20;30(2):99. doi: 10.1007/s11325-026-03646-7 (PMC13002775; doi:10.1007/s11325-026-03646-7)

**
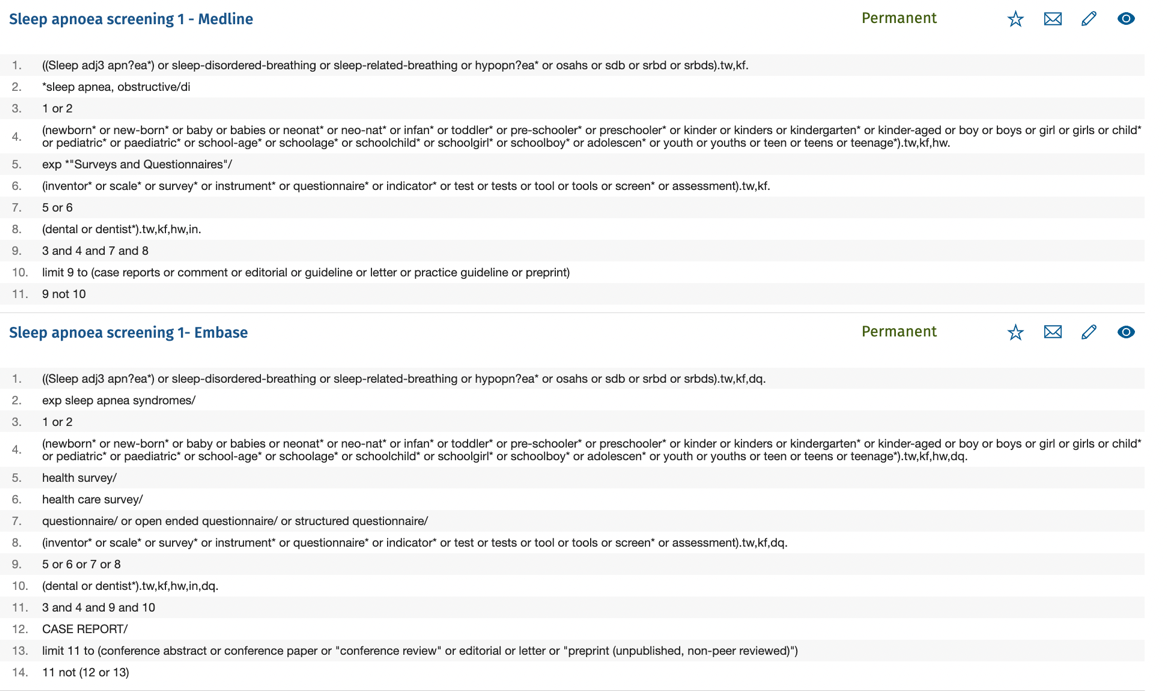
**

**
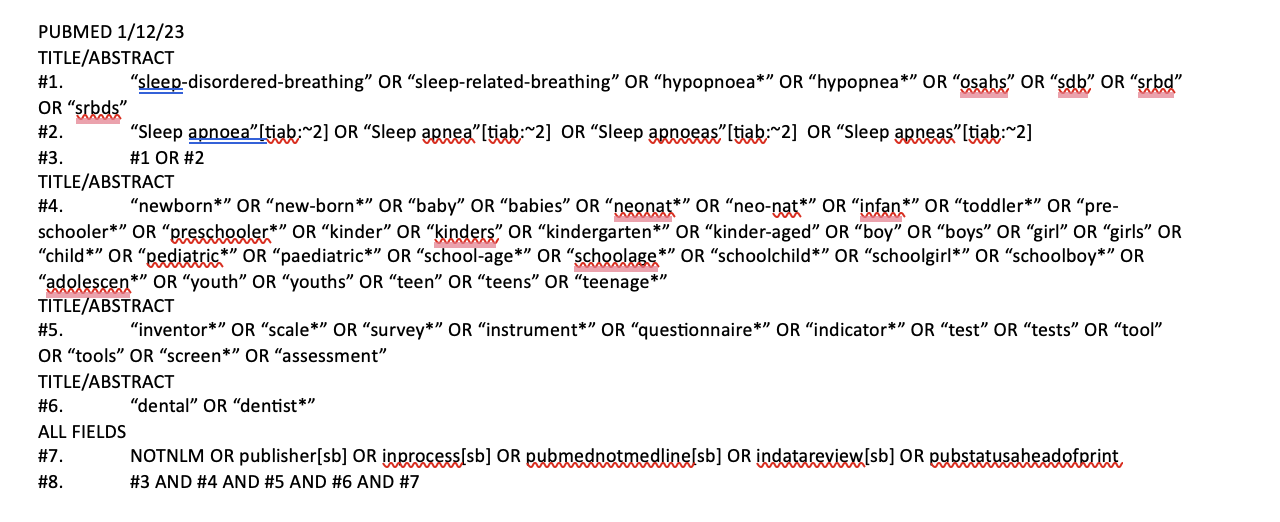
**

Supplement: Supplementary file 2 — Supplementary Material 2 (DOCX 689 KB) [file 11325_2026_3646_MOESM2_ESM.docx]
