# Supplementary material for: Screening for paediatric sleep disordered breathing in the dental setting: a scoping review
Source: Sleep Breath. 2026 Mar 20;30(2):99. doi: 10.1007/s11325-026-03646-7 (PMC13002775; doi:10.1007/s11325-026-03646-7)
Supplement: Supplementary file 3 — Supplementary Material 3 (DOCX 65.0 KB) [file 11325_2026_3646_MOESM3_ESM.docx]

| Title | Year | Authors | Study design | Country | Aim | Age Range | Sample Size | Gender Distribution | Setting | Screening Tool Described | Outcome Variables | Results | Proportion of Patients + SDB risk | Referral Pathway Described | Referral Type |
| --- | --- | --- | --- | --- | --- | --- | --- | --- | --- | --- | --- | --- | --- | --- | --- |
| Parents‘ Perceptions of Breathing Pattern Changes, Sleep Quality, and Fatigue in Children after Rapid Maxillary Expansion: A Survey and Case Series Study | 2019 | Narmin Helal, Osama Basri, Lubabah Samir Gadi, Amira Fahad Alhameed and John Marshal Grady | Mixed methods prospective cohort study | USA | Assess parent-reported changes in sleep/breathing after RME, plus nasal changes via CBCT in 6 cases | 5 to 13 | 116 | 49 boys, 67 girls | Private orthodontic practice in Pittsburgh, Pennsylvania, USA | PSQ | Parents’ perception of changes in breathing patterns, sleep quality, fatigue and behaviour | 59% improvement in PSQ items; >80% drop in some snoring symptoms; mean nasal cavity area changes were 4.1 mm² |  | No |  |
| Snoring and sleep disturbance among children from an orthodontic setting. | 2001 | Nelson, S; Kulnis, R | Cross sectional study | USA | Assess prevalence of snoring & identify sleep behaviour patterns associated with increased odds of snoring. | 6 to 17 | 405 | 219 females, 186 males | School of Dentistry and from several private practices in Cleveland, Ohio. | Other: Sleep questionnaire Ali NJ, Pitson DJ, Stradling JR. (1993) | Questionnaire responses, Odds of snoring | 17% habitual snorers; Increased odds of snoring with mouth breathing, sleeping with head tipped back, morning headaches, frequent coughs or colds | 17% (snoring) | No |  |
| The correlation between craniofacial morphology and sleep-disordered breathing in children in an undergraduate orthodontic clinic. | 2011 | Tsuda, Hiroko; Fastlicht, Sandra; Almeida, Fernanda R; Lowe, Alan A | Cross sectional study | Canada | To determine the relationship between sleep-disordered breathing (SDB) symptoms and craniofacial morphology | >6 (primary or mixed dentition) | 173 | 87 males and 86 females | Undergraduate program at the University of British Columbia | OSA-18 | OSA-18 results, cephalometric measurements, study model measurements | 1.1% had increased SDB risk; >20% reported loud snoring, mouth breathing, or trouble waking. Higher scores linked to retroclined incisors, high palate (late mixed dentition), long soft palate (early mixed dentition) | 1.10% | Yes | Sleep specialist |
| Associations between sleep-disordered breathing symptoms and facial and dental morphometry, assessed with screening examinations. | 2011 | Nelly T Huynh, Paul D Morton, Pierre H Rompr, Athena Papadakis, Claude Remise | Cross sectional study | Canada | To evaluate the prevalence of sleep-disordered breathing symptoms and their associations with facial or dental morphometry in a general paediatric orthodontic population. | <18 | 604 | 335 females and 269 males | General orthodontic department of a university clinic (Universite de Montreal in Canada) | PSQ; Other: Pittsburgh Sleep Quality Index | Facial and dental morphometry and SDB symptoms | Prevalence of snoring (2.9–10.9%), mouth breathing (34–36%), sleepiness (3.3–23.6%), behavioural issues (7.7–26.7%). Associated with adenotonsillar hypertrophy, craniofacial features (e.g., dolichofacial pattern, narrow palate), and allergies | 1.8-36.1% (one or more SDB symptoms) | No |  |
| Sleep-disordered breathing in orthodontic practice: Prevalence of snoring in children and morphological findings. | 2016 | Graf, Isabelle; Schumann, Uwe; Neuschulz, Julia; Hofer, Karolin; Ritter, Lutz; Braumann, Bert | Cross sectional study | Germany | To evaluate the prevalence of snoring and its correlation with craniofacial/upper airway morphology in young individuals with orthodontic treatment need. | 7 to 15 | 100 | 54 girls, 46 boys | Private Orthodontic practice in Germany | Other: Questionnaire used not specified (8 questions) | Snoring, Airway morphology (PAS dimensions) | Snoring reported in 53% of children (63% boys, 44% girls). Snorers had significantly reduced posterior airway space (PAS) compared to non-snorers. Larger SNA angles were associated with increased sagittal PAS dimensions at the maxillary level. | 53% (snoring) | No |  |
| Sleep disordered breathing in children seeking orthodontic care | 2018 | Rohra, Ashok K Jr; Demko, Catherine A; Hans, Mark G; Rosen, Carol; Palomo, Juan Martin | Cross sectional study | USA | To evaluate the prevalence of positive or potential SDB in children in the orthodontic population. | 9 to 17 | 303 | 152 males, 151 females | Orthodontic clinic, Cleveland Ohio | PSQ | PSQ score, demographic data | High-risk status on the PSQ was not associated with sex, age, or race. The percentage of patients who were screened as high risk was 7.3%. | 7.30% | Yes | Paediatric Sleep Medicine |
| Upper Airway Changes following Functional Treatment with the Headgear Herbst or Headgear Twin Block Appliance Assessed on Lateral Cephalograms and Magnetic Resonance Imaging. | 2019 | Gu, Min; Savoldi, Fabio; Hagg, Urban; McGrath, Colman P J; Wong, Ricky W K; Yang, Yanqi | Randomised controlled trial | Hong Kong | To examine whether HG-Herbst or to the HG-TB treatment show differences in upper airway dimension changes or sleep disordered breathing changes. | 12-17 years old for boys and 10-15 years old for girls (ages at which the pubertal growth spurt occurs) | 28 | 11 boys, 17 girls | Faculty of Dentistry at University of Hong Kong | PSQ | Upper airway dimensions (MRI and Lateral Cephalogram), SDB (PSQ) | No significant differences were observed in the upper airway structures or SRBD between the two groups. |  | No |  |
| Orthodontic Removable Appliance with Posterior Bite Plane Use is not Associated with Developing Sleep-Disordered Breathing Symptoms in Healthy Children. | 2019 | Changsiripun, Chidsanu; Tokavanich, Nicha; Almeida, Fernanda R | Cohort study: Longitudinal observational study | Thailand | To assess the development of sleep disordered breathing (SDB) symptoms and the relationship between the amount of increased vertical dimension and severity of SDB symptoms in healthy children wearing orthodontic removable appliances with posterior bite planes. | 8 to 12 | 16 | 8 males and 8 females | Faculty of Dentistry, Chulalongkorn University, Bangkok Thailand | PSQ; OSA-18 | OSA-18 and PSQ results | There were no significant differences between the OSA-18 or PSQ results collected one-month preinsertion of the appliance; one day post-insertion; and one month post-insertion. No significant relationship between the posterior bite plane thickness and the difference in scores. |  | No |  |
| Oral health in children with sleep-disordered breathing: a cross-sectional study. | 2019 | Grillo, Calogero; La Mantia, Ignazio; Zappala Graziano; Cocuzza, Salvatore; Ciprandi, Giorgio; Andaloro, Claudio | Cross sectional study | Italy | To investigate the oral health status and oral health-related quality of life (OHRQoL) in children at risk of SDB compared with a control group not at risk for SDB. | 8 to 17 | 122 | 64 males, 58 females | Dental clinic of the Catania University | PSQ | SDB risk, DMFS: decayed, missing, and filled surfaces (for permanent and primary teeth); COHIP: Child Oral Health Impact Profile; PPD: probing pocket depth; BOP: bleeding on probing | There was a significant association between SDB and all six outcomes with higher values in SDB+ children. SDB+ was associated with a poorer OHRQoL, and a greater COHIP score for both parents and children. |  | No |  |
| Pediatric sleep-disordered breathing in the orthodontic population: Prevalence of positive risk and associations | 2020 | Abtahi, Sahar; Witmans, Manisha; Alsufyani, Noura A; Major, Michael P; Major, Paul W | Cross sectional study | Canada | To determine the prevalence of overall SDB risk, habitual snoring, and sleepiness in the orthodontic population, and compare it to that previously reported in a paediatric population. | 5 to 16 | 390 | 173 Males, 217 females | University of Alberta clinic and 9 other private practices in Alberta | PSQ | Positive Risk of SDB (PSQ), Medical comorbidities | SDB risk prevalence was 10.8% in orthodontic patients, higher than 5% in healthy children. Snoring (13.3%) and sleepiness (17.9%) were common. Higher SDB risk was associated with increased rates of nocturnal enuresis (13.6%), high BMI (18.2%), and ADHD (31.8%). | 10.80% | Yes | Not specified |
| Correlation between Parental-Reported Tooth Grinding and Sleep Disorders: Investigation in a Cohort of 741 Consecutive Children | 2020 | M. Segu, M. Pollis, A. Santagostini, F. Meola, and D. Manfredini | Cross sectional study | Italy | To evaluate the correlation between parent reported symptoms of sleep bruxism and other symptoms/signs of sleep disorders. | 8 to 12 | 741 | 409 males, 332 females | Private orthodontic clinic | SDSC | Parental reported tooth grinding and sleep disorder signs (SDSC results) | Association noted between parental-reported tooth grinding and sleep disorder signs i.e., bedtime problems, night awakenings, nocturnal symptoms, and morning symptoms. |  | No |  |
| Prevalence of Sleep-Disordered Breathing in Children Referring for First Dental Examination. A Multicenter Cross-Sectional Study Using Pediatric Sleep Questionnaire | 2020 | Di Carlo, Gabriele; Zara, Francesca; Rocchetti, Milena; Venturini, Angelica; Ortiz-Ruiz, Antonio Jose; Luzzi, Valeria; Cattaneo, Paolo Maria; Polimeni, Antonella; Vozza, Iole | Cross sectional study | Italy and Spain | To determine the prevalence of sleep disordered breathing in a population referring for first dental examination to paediatric dental services and investigate  correlation between general health status recorded from the first visit anamnestic data and sleep-disordered breathing | 2 to 16 | 668 | 328 males, 323 females, 17 unknowns | Department of Oral and Maxillo-Facial Sciences, Sapienza University of Rome, Italy and Unit of Integrated Paediatric Dentistry, University of Murcia, Spain. | PSQ | SDB risk (PSQ score) | The prevalence of SRDB was 9.7% for the sample. There was a positive correlation between gender, snoring, bad habits, anxiety and SRDB. | 9.70% | No |  |
| Opportunity for Interprofessional Collaboration: Screening for Pediatric Sleep-Disordered Breathing by Dentists | 2020 | Okuji, David; Healy, Elodi; Wu, Yinxiang | Cross sectional study | USA | To identify the associations between paediatric subjects, who respond positively to the Paediatric Sleep Questionnaire, (i.e.eight or more items on the PSQ), and their sociodemographic and clinical findings. | 2 to 18 | 1000 | 462 females, 538 males | Approved training locations with the Postdoctoral Dental Residency Programs at NYU (Arizona, Florida, Hawaii, Maryland Massachusetts, New York, and Tennessee centers) | PSQ | PSQ score, sociodemographic and clinical findings, such as American Society of Anesthesiologists (ASA) status, attention deficit hyperactivity disorder (ADHD), obesity, age, and anterior overjet. | 11.9% had a PSQ score of at least eight. ASA status, anterior overjet, attention deficit hyperactivity disorder, and obesity were significant predictors of the presence of a PSQ score of at least eight. | 11.90% | No |  |
| Determinants of probable sleep bruxism in a pediatric mixed dentition population: a multivariate analysis of mouth vs. nasal breathing, tongue mobility, and tonsil size | 2020 | Oh, James S; Zaghi, Soroush; Peterson, Cynthia; Law, Clarice S; Silva, Daniela; Yoon, Audrey J | Cross sectional Study | USA | To identify parent-reported measures and clinical findings associated with probable sleep bruxism (PSB) among children. The secondary objective of this study was to determine the association between the various factors through multivariate exploratory analysis | 6 to 12 | 96 | 46 males, 50 females | UCLA Childrens Dental Center, Los Angeles, California, USA | SDSC; Other: FAIREST-15 | Parental reporting of PSB and sleep symptoms, Clinical assessment of tonsil hypertrophy, tongue mobility, and nasal obstruction. | 24% of children met diagnostic criteria for PSB. Those with PSB had higher SDSC scores, indicating greater risk for sleep disturbances. PSB was significantly linked to impaired nasal breathing, habitual mouth breathing, restricted tongue mobility, and tonsillar hypertrophy. | 24% (probable sleep bruxism) | No |  |
| Potential impact of pediatric obstructive sleep apnea on mandibular cortical width dimensions | 2021 | Fernandes Fagundes, Nathalia Carolina; d'Apuzzo, Fabrizia; Perillo, Letizia; Puigdollers, Andreu; Gozal, David; Graf, Daniel; Heo, Giseon; Flores-Mir, Carlos | Cross sectional study | Canada, Italy, Spain | To analyse differences in MCW among children either diagnosed with OSA by nocturnal polysomnography (nPSG) or identified as at high-risk or low risk for OSA based on the Paediatric Sleep Questionnaire (PSQ). | <18 | 161 | 96 males, 65 females | Orthodontic and Sleep Clinic at the University of Alberta, Edmonton (Canada), the Orthodontic Program at the University of Campania Luigi Vanvitelli in Naples (Italy), and the Department of Orthodontics of the International University of Catalonia in Barcelona (Spain) | PSQ | Mandibular cortical width dimensions | The PSG diagnosed OSA group had smaller MCW than the low-risk OSA group. The high-risk PSQ OSA group had smaller MCW than the low-risk group. There was no difference between the PSG diagnosed and high-risk PSQ group. (polysomnographically diagnosed OSA vs high-risk OSA: P =.085) |  | No |  |
| Nasal cavity structural anomalies in children and adolescents at high risk of sleep-disordered breathing: An exploratory cone-beam computed tomography study | 2019 | Baddam, Pranidhi; Thereza-Bussolaro, Claudine; Flores-Mir, Carlos; Graf, Daniel | Cross sectional study | Canada | Investigate the presence of structural anomalies in the nasal cavity (deviated nasal septum [DNS] and turbinate hypertrophy [TH]) in patients at high risk or not of sleep-disordered breathing (SDB). | 6 to 17 | 99 | 57 males, 42 females | Graduate Orthodontic Program Clinic at the University of Alberta | PSQ | SDB risk (PSQ score), deviated nasal septum [DNS] and turbinate hypertrophy [TH] | The prevalence of a PSQ score suggestive of a high risk of SDB in this sample was 59%. Only the presence of subjectively determined TH in patients is associated with a high risk for SDB (positive PSQ). | 59% | No |  |
| Determinants of Sleep-Disordered Breathing During the Mixed Dentition: Development of a Functional Airway Evaluation Screening Tool (FAIREST-6). | 2021 | Oh, James S; Zaghi, Soroush; Ghodousi, Nora; Peterson, Cynthia; Silva, Daniela; Lavigne, Gilles J; Yoon, Audrey J | Cross sectional study | USA | To identify functional, extraoral, and intraoral features associated with increased risk of sleep disturbances in paediatric patients to develop a functional airway screening tool for early diagnosis of paediatric SDB. | 6 to 12 | 96 | 46 males, 50 females | UCLA Children‚Dental Center, Los Angeles, California | SDSC; Other: FAIREST-6 | Functional, extraoral, and intraoral features associated with increased risk of sleep measurements in paediatric patients (determined by higher SDSC scores) | Mouth-breathing, mentalis strain, tonsillar hypertrophy, ankyloglossia, dental wear, and narrow palate were key clinical signs linked to higher SDSC scores. These signs formed the basis of the validated FAIREST-6 screening tool. |  | No |  |
| Tonsil Size and Mallampati Score as Clinical Predictive Factors for Obstructive Sleep Apnea Severity in Children. | 2021 | Zreaqat, Maen; Hassan, Rozita; Samsudin, A R; Stas, Yasser; Hanoun, Abdulfatah | Cross sectional study | Malaysia | To determine differences between OSA and non-OSA groups in terms of BMI, tonsil size and, Mallampati score | 11 to 14 | 164 | 95 males, 69 females | Private orthodontic clinic | PSQ; Other: Tonsil size, mallampati score | OSA, BMI, Tonsil size, Mallampati score | BMI, tonsil size and the Mallampati score was higher in the OSA group compared to controls. A correlation was recorded between the Mallampati score and OSAS severity, but not with BMI or tonsil size. |  | No |  |
| Evaluation of the relationship between malocclusions and sleep-disordered breathing in children. | 2022 | Duma, Sacide; Vural, Handan | Cross sectional study | Turkey | To assess the prevalence of high-risk SDB in the sample, and the relationship between malocclusions and SDB. | 7 to 15 | 240 | 108 males, 132 females | Pedodontics Department of a Faculty of Dentistry in Turkey | PSQ | SDB risk (PSQ score), Dental/Skeletal malocclusions (clinical assessment) | 25.8% children were in the high-risk group. The children in this group mostly had a convex profile (48.4%), mandibular retrognathia (54.8%) and high-angle growth direction (54.8%). The prevalence of habitual snoring, mouth breathing, and dry mouth was 48.4%, 64.5%, and 87.2% amongst high-risk children. | 25.80% | Yes | Orthodontist, Otolaryngologist |
| Efficacy of Preformed Sleep and Habit Appliances to Modify Symptoms of Sleep-Disordered Breathing and Oral Habits in Children with Focus on Resolution of Mouth Breathing. | 2022 | Bergersen, Earl O; Stevens-Green, Brooke; Rosellini, Elizabeth | Cohort study: longitudinal observational study | USA | To determine the potential therapeutic benefit of a preformed oral appliance to eliminate or reduce poor oral habits and symptoms associated with sleep-disordered breathing. | 2 to 13 | 220 | 110 males, 110 females | Private dental practices in USA | Other: SDB Questionnaire for children (2015) | Symptoms of SDB | With passive night-time wear of the preformed appliance, 75% of total symptoms measured experienced improvement, mean improvement of 76%. 0.3% of all observed symptoms increased in severity, and 24% had no change. |  | Yes | Sleep clinic/ENT if patient unable to breathe through nose/Stage 3 or 4 Friedman's Grade Tonsils |
| Sleep-disordered breathing in children and adolescents seeking paediatric dental care in Dubai, UAE | 2021 | Abdalla, M; Halabi, M; Kowash, M; Hussein, I; Khamis, A; Salami, A | Cross sectional study | UAE | To evaluate the prevalence of SDB risk amongst UAE children and adolescents, identify the association between BMI, prematurity, malocclusion, hypomineralisation, tonsil size and SDB risk. | 7 to 16 | 65 | 36 males and 29 females | Paediatric Dentistry Department at Dubai Dental hospital | PSQ | Positive risk of SDB (PSQ), Tonsil size, BMI, Occlusion | 12.3% of children at high-risk of SDB. Risk of SDB associated with tonsil size, BMI, Class-II molars, and posterior crossbite. Enamel defects, overjet and premature birth not statistically related to the high risk. | 12.30% | Yes | Paediatric Sleep Medicine Specialist |
| Effect of Twin-block Appliance on Pharyngeal Airway, Sleep Patterns, and Lung Volume in Children with Class II Malocclusion. | 2022 | Batra, Akriti; Shetty, Vabitha | Cohort study: longitudinal observational study | India | To evaluate the effect of the twin-block appliance on pharyngeal airway dimensions, sleep patterns, and lung volumes in growing children with class II malocclusion with a retrognathic mandible | 9 to 12 | 20 | 11 males, 9 females | Outpatient Department of Paediatric and Preventive Dentistry, AB Shetty Memorial Institute of Dental Sciences, Deralakatte, Mangaluru | Other: Sleep questionnaire Yerra, A & Shetty, V (2021) | Pharyngeal airway dimensions, sleep patterns, and lung volumes (spirometry changes) | Twin block treatment improved sleep problems such as snoring, noisy breathing, and mouth breathing. Pharyngeal airway dimensions, and soft palate length and thickness also improved. Lung volume increased but not significantly. |  | No |  |
| Mallampati and Brodsky Classification and Children's Risk for Sleep Related Breathing Disorder | 2022 | Lesavoy, Bret; Lumsden, Christie; Grunstein, Eli; Yoon, Richard | Cross sectional study | USA | To evaluate associations between Mallampati and Brodsky classification and children‚ risk for sleep related breathing disorder (SRBD) | 2 to 11 | 150 | 76 females, 74 males | Columbia University community dental clinic in New York City | PSQ | SDB risk (PSQ score) | Children with Mallampati class III and IV and Brodsky grade 3 and 4 collectively had a 5.24-fold and 2.8-fold increase in SRBD risk compared to children with class I and II and grade 1 and 2, respectively. |  | No |  |
| Sleep-disordered breathing in children seeking orthodontic care-an Australian perspective. | 2023 | Wellham, A; Kim, C; Kwok, S S; Lee, Rjh; Naoum, S; Razza, J M; Goonewardene, M S | Cross sectional study | Australia | To determine the prevalence of children at risk of sleep-disordered breathing in an Australian orthodontic population | 4 to 18 | 1209 | 653 female, 556 males | Two private orthodontic practices in Perth, Western Australia | Other: Modified PSQ (Modified to 21 questions with binary (yes/no) responses) | SDB risk (PSQ score) | 7.3% were at risk of sleep-disordered breathing. An association between sex and the potential risk of sleep-disordered breathing was found with 11% of males at risk of sleep-disordered breathing compared to 7% of females. | 7.30% | No |  |
| Prevalence of sleep-related breathing disorders in children with malocclusion | 2020 | Vazquez-Casas, Ivette; Sans-Capdevila, Oscar; Moncunill-Mira, Jordi; Rivera-Baro, Alejandro | Cohort study: longitudinal observational study | Spain | To survey the prevalence of SRBD in patients presenting for orthodontic treatment | Children with an average age of 8.5 years | 249 | 123 girls and 126 boys. | Orthodontic department of Sant Joan de Du Hospital | PSQ; SDSC | SRBD, Dentofacial characteristics | 22.8% of children had SRBD, with higher prevalence in those with adenotonsillectomy. No significant link found between SRBD and dentofacial traits. No significant change in SDSC scores post-treatment. | 22.80% | No |  |
| Sleep Difficulties and Symptoms of Attention-deficit Hyperactivity Disorder in Children with Mouth Breathing | 2021 | Kalaskar, Ritesh; Bhaje, Priyanka; Kalaskar, Ashita; Faye, Abhijeet | Cross sectional study | India | To assess sleep disturbances and ADHD symptoms in children having mouth breathing. | 7 to 12 | 100 | 70 males, 30 females | Department of pedodontics and preventive dentistry, Government Dental College and Hospital, Maharashtra, India | Other: Children sleep habits questionnaire (CSHQ) | CSHQ results and ADHD symptoms (interview results) | CSHQ showed highest scores in SDB, sleep onset delay, and daytime sleepiness. Sleep duration and daytime sleepiness were negatively correlated with hyperactivity; daytime sleepiness was positively correlated with inattention (p < 0.01). 7 participants satisfied criteria for ADHD. . |  | No |  |
| Sleep-Related Breathing Disorders in Children-Red Flags in Pediatric Care. | 2022 | Blumer, Sigalit; Eli, Ilana; Kaminsky-Kurtz, Shani; Shreiber Fridman,Yarden;Dolev, Eran; Emodi-Perlman,Alona | Cross sectional study | Israel | To determine anamnestic and clinical findings that can serve as red flags indicating the presence of SRBD in children. | 4 to 12 | 227 | 113 females, 114 males | Department of Paediatric Dentistry, School of Dental Medicine, Tel Aviv University | PSQ | SDB risk (PSQ), oral behaviours and habits, general health status, parental self-report | SDB risk prevalence: 10.6%. Children with SDB had more developmental delay, non-continuous sleep, mouth breathing, and snoring. Odds of SDB were significantly increased by ADHD medication use (×7), non-continuous sleep (×6), mouth breathing (×5), and snoring (×3). | 10.60% | No |  |
| Management of the Pediatric Patient with Suspected Diagnosis of Obstructive Sleep Apnea Syndrome | 2023 | Sorina Savin, Luca Mezzofranco, Antonio Gracco, Giovanni Bruno and Alberto De Stefani | Longitudinal Observational descriptive study | Italy | To describe the multidisciplinary management pathway for paediatric patients with suspected obstructive sleep apnea syndrome (OSAS) conducted by the Paediatric and Orthodontic Department of the Dental Clinic of Padua | Paediatric and Adolescent patients- age range not specified | 134 | 76 males, 58 females | Dental Clinic of Padua, Department of Pedodontics and Orthodontics | PSQ | Number of diagnostic suspect patients that tested positive based on respiratory polygraphic results, and their multidisciplinary management pathway | Of the 134 patients identified as diagnostic suspects, 38 patients (28.3%) tested positive based on respiratory polygraphic results. Depending on the severity, patients were referred to various specialists (dietician, maxillofacial team, pulmonologist, otolaryngologist) | 28.3% (respiratory polygraphic testing) | Yes | ENT (Ear, Nose, and Throat) specialists, nutritionists, and speech therapists, depending on the severity and problem |
| Association of Sleep-Disordered Breathing and Developing Malocclusion in Children: A Cross-Sectional Study. | 2023 | Shirke, Shweta R; Katre, Amar N | Cross sectional study | India | To assess the prevalence of SDB and its association with developing malocclusion in six to 12-year-old children and the effect of modifiers like age, gender, and tonsillar enlargement. | 6 to 12 | 161 | 75 males, 86 females | Outpatient department of Department of Paediatric and Preventative Dentistry, Yerala Medical Trust (YMT) Dental College and Hospital, Navi Mumbai, IND | PSQ | Primary outcomes: SDB score, Angle class of malocclusion, and IOTN (index of orthodontic treatment need) grade. Modifying variables: age, gender, and tonsillar enlargement (Brodsky criteria). | SDB prevalence was 69%. SDB significantly associated with angle class II and class III malocclusion and with higher IOTN grades. Logistic regression revealed that gender and tonsillar enlargement had a significant modifying effect. | 69% | No |  |
| Prevalence of Sleep-Disordered Breathing in Children Ages Seven to 10 Years: A Comparative Study Before and After COVID-19 Lockdown. | 2023 | Burnheimer, John M; Loo, Brenda H; Kaufer, Jamie A | Cross sectional study | USA | To investigate the prevalence of positive or potential SDB in a paediatric population pre- and post-COVID-19 lockdown, and determine the association of other risk factors with SDB risk. | 7 to 10 | 366 | 183 males, 183 females | Two private paediatric dental practices in Southwestern Pennsylvania | PSQ | Positive risk of SDB, Gender, BMI | 16.2 percent were high risk of SDB post Covid-19 lockdown in contrast to 5.0 percent pre lockdown. There were no associations between sex or body mass index and high risk of SDB. | 16.20% | No |  |
| Efficacy of Roncolab mobile application for diagnosing the primary sign of sleep-disordered breathing (snoring) in children | 2023 | Aragon-Villalba G, Munoz-Quintana G, Rubin de Celis GN, Torres Hortelano JM, Espinosa de Santillana IA. | Cross sectional study | Mexico | To compare the RoncoLab app and the PSQ regarding how efficiently they diagnose snoring | 7 to 11 | 31 | 20 boys and 11 girls | Paediatric dental clinic at BUAP Hospital, Mexico | PSQ | Kappa index for determination of primary snoring between the PSQ and the Roncolab app, Roncolab app sensitivity and specificity. | The Kappa index for identification of primary snoring was 0.743 (p<0.05). App sensitivity was 0.92, and specificity 0.82 | 51.6% (Primary snoring Roncolab app), 45.2% (Primary snoring PSQ) | No |  |
| Association among craniofacial morphology, ethnicity, and risk of pediatric sleep-related breathing disorders: A multicenter study. | 2023 | Kim, Chai Yoon; Reinertsen, Erik; Dang, Calvin; Nkutshweu, Dineo; Sathekge, Rachel; Choi, Yoon Jeong; Cha, Jung-Yul; Alturki, Ghassan; Jamel, Ahmad; Suzuki, Akihiro; Arai, Kazuhito; Amm, Elie; Motro, Melih; Parsi, Goli | Cross sectional study | USA, South Africa, South Korea, Saudi Arabia, and Japan | To investigate the associations between SRBD risk (as assessed by PSQ score) and craniofacial anatomic features (as assessed by cephalometric measurements) in children of different ethnicities (white, African, Hispanic, Asian, and Middle Eastern) from 5 global clinical sites. | 5 to 18 | 602 | 341 females, 261 males | Boston (Boston, Mass), Military Hospital (Pretoria, South Africa), Yonsei University (Seoul, South Korea), Nippon Dental University (Tokyo, Japan), and King Abdul Aziz University (Jeddah, Saudi Arabia). | PSQ | PSQ score and cephalometric measurements | Male gender, Middle Eastern ethnicity, body mass index, gonial angle, and inferiorly positioned hyoid were significantly associated with the risk of SRBD. |  | No |  |
| Malocclusions, Sleep Bruxism, and Obstructive Sleep Apnea Risk in Pediatric ADHD Patients: A Prospective Study. | 2024 | Alessandri-Bonetti, Anna; Guglielmi, Federica; Deledda, Giulia; Sangalli, Linda; Brogna, Claudia; Gallenzi, Patrizia | Cross sectional | Italy | To investigate the prevalence of OSA risk in children with ADHD, as well as the prevalence of malocclusions and sleep bruxism, and to examine possible association with OSA. | 6 to 16 | 80 | 62 Males and 18 females | Paediatric dental clinic at the Paediatric Dentistry Unit of Fondazione Policlinico Universitario, IRCCS, Rome | PSQ | OSA risk, malocclusion, sleep bruxism | Children with ADHD showed a higher prevalence of OSA risk (62.5%) and probable sleep bruxism (40%) compared to controls (10% and 7.5%, respectively). No significant differences were found in malocclusion rates between groups. | 62.5% (ADHD group) 10% (control group) | No |  |
| Sleep disordered breathing and oral health-related quality of life in children with different skeletal malocclusions | 2022 | Coban, Gokhan; Buyuk, S Kutalmis | Cross sectional study | Turkey | To evaluate the relationship among different skeletal malocclusion patterns, sleep disordered breathing (SDB) and children‚ oral health-related quality of life (OHRQoL). | 8 to 15 | 205 | 101 boys, 104 girls | Faculty of Dentistry, Ordu University | PSQ | PSQ results and OHIP-14 scores  (oral health impact) | SDB in 10.7% of children. Prevalence of snoring, breathing difficulty during sleep, mouth breathing, and dry mouth on awakening were 8.78%, 7.31%, 36.09%, and 37.07%, respectively. Positive correlation between OHIP-14 and PSQ scores in children with Class I and III malocclusion. | 10.70% | No |  |
